# PERCEIVED PHYSICAL LITERACY FOR CHINESE ELDERLY QUESTIONNAIRE DEVELOPMENT: PRELIMINARY VALIDITY AND RELIABILITY

**DOI:** 10.1093/geroni/igz038.1927

**Published:** 2019-11-08

**Authors:** Haocen Wang, Barbara King

**Affiliations:** University of Wisconsin-Madison, Madison, Wisconsin, United States

## Abstract

Physical activity (PA) is an essential health prompting behavior. Unfortunately, in China, only about 12% to 40% of older adults met the PA recommendations. To guide the design of future PA interventions and policy-making, innovative solutions are needed to be introduced. Physical literacy, a relatively novel concept, has been recently introduced in the field of older adults’ PA. This concept takes a holistic view of PA behavior, which proposed that a person need to be motivationally, physically, strategically, effectively, socially, and knowledgeably prepared to be and stay physically active. The aim of this study was to develop the Perceived Physical literacy for Chinese Elderly (PPLCE) questionnaire and to establish its reliability and validity. An item pool for the PPLCE was generated from literature and interviews with Chinese older adults. Expert panel reviews and cognitive interviews were applied to establish the face and content validity of the questionnaire. A convenient sample of 388 Chinese older adults was recruited to assess the psychometric properties of the PPLCE. The item-level analysis and exploratory factor analysis resulted in a 46-item self-report measure, consisting with four factors: motivation, physical competence, interaction with environment, sense of self, interaction with others, and knowledge and understanding. The Cronbach’s alpha coefficient and test-retest reliability of the PPLCE were 0.88 and 0.79 respectively. A positive correlation between PPLCE and leisure-time PA was found (r=0.43). The PPLCE has the potential to be used as a valid and reliable measure to assess Chinese older adults’ perceived physical literacy.

